# Purification and Characterization of Recombinant Botulinum Neurotoxin Serotype FA, Also Known as Serotype H

**DOI:** 10.3390/toxins10050195

**Published:** 2018-05-11

**Authors:** Gavin Hackett, Kevin Moore, David Burgin, Fraser Hornby, Bryony Gray, Mark Elliott, Imran Mir, Matthew Beard

**Affiliations:** Ipsen Bioinnovation Ltd., 102 Park Drive, Milton Park, Abingdon OX14 4RY, UK; gavin.s.hackett@gmail.com (G.H.); kevin.moore@ipsen.com (K.M.); dave.burgin@ipsen.com (D.B.); fraser.hornby@ipsen.com (F.H.); bryony.gray@ipsen.com (B.G.); mark.elliott@ipsen.com (M.E.); imran.mir.abingdon@ipsen.com (I.M.)

**Keywords:** bacterial toxin, botulinum toxin, neurotoxin, neurotransmitter release, protein purification, proteolytic enzyme, recombinant protein expression, SNARE proteins

## Abstract

We have purified and characterized recombinant botulinum neurotoxin serotype FA (BoNT/FA). This protein has also been named as a new serotype (serotype H), but the classification has been controversial. A lack of well-characterized, highly pure material has been a roadblock to study. Here we report purification and characterization of enzymatically active, and of inactive nontoxic, recombinant forms of BoNT/FA as tractable alternatives to purifying this neurotoxin from native *Clostridium botulinum*. BoNT/FA cleaves the same intracellular target proteins as BoNT/F1 and other F serotype BoNTs; the intracellular targets are vesicle associated membrane proteins (VAMP) 1, 2 and 3. BoNT/FA cleaves the same site in VAMP-2 as BoNT/F5, which is different from the cleavage site of other F serotype BoNTs. BoNT/FA has slower enzyme kinetics than BoNT/F1 in a cell-free protease assay and is less potent at inhibiting ex vivo nerve-stimulated skeletal muscle contraction. In contrast, BoNT/FA is more potent at inhibiting neurotransmitter release from cultured neurons.

## 1. Introduction

Botulinum neurotoxins (BoNTs) are highly potent neurotoxic proteins produced by various species of *Clostridia* bacteria [1]. Conventionally, BoNTs are classified into seven different families, called serotypes, based on their sensitivity to neutralization by various reference antisera. The serotype families are: BoNT/A, B, C1, D, E, F, and G [1,2,3]. Increasingly, BoNTs are classified into these serotype groups based on sequence alignment, which clusters them into phylogenetic groups, rather than by antibody neutralization experiments. Classification by sequence alignment correlates well with experimentally determined serotype assignments [4] and also highlights a number of mosaic neurotoxins, which contain domains that align into different phylogenetic groups from each other; examples include BoNT/CD [5] and BoNT/DC [6].

The structural and functional domains that make up BoNTs are well-conserved [7]. BoNTs are expressed as single chain proteins that become post-translationally cleaved into their mature heterodimeric form. This comprises a single domain light chain subunit (LC), which is approximately 50 kDa, and a three-domain heavy chain subunit (HC), which is approximately 100 kDa. Cleavage into the heterodimeric form is called activation and increases the neurotoxic activity. The site of cleavage, which is called the activation loop, is bounded by cysteine residues that form a disulphide bridge and covalently link the light and heavy chains. Key functional steps in the neurotoxic mechanism of action directly map to individual domains [8]. The LC is a specific protease that cleaves soluble *N*-ethyl-maleimide-sensitive factor attachment protein receptor proteins (SNARE proteins) inside target neurons [9]. The most N-terminal of the HC domains (Hn) contains a transmembrane translocation activity that transports the LC protease into the neuronal cytoplasm [10,11]. The most C-terminal HC domain (Hcc) contains neuron-specific high-affinity binding activity [12,13]. The domain located between Hn and Hcc (called Hcn) is of unknown function but may be involved in binding lipids [14,15].

Most BoNT producing strains of *Clostridia* express just one serotype. However, some strains produce more than one [16]. Strains that produce two serotypes are called bivalent strains [17]. Bivalent strains often express different amounts of each serotype with relatively high expression of one serotype, called the major toxin, and lower expression of the other, called the minor toxin. Conventionally, upper and lower case letters designate major and minor toxins respectively. For example, CDC4013 is a Bf bivalent strain which produces high levels of a B serotype and relatively low levels of an F serotype BoNT [18].

BoNT/FA, is a mosaic neurotoxin and is the minor toxin produced by the bivalent strain IBCA 10-7060 [19,20], which has also been subcultured and named CDC69016 [21]. The major toxin in this strain is a B2 serotype [19,21,22]. Initially, the minor toxin BoNT/FA was designated as a new serotype (BoNT/H) because it appeared relatively insensitive to reference antisera [19]. However, subsequent studies report that a heptavalent botulism antitoxin (Equine BAT; Emergent BioSolutions, Rockville, Maryland, USA) can neutralize the activity of the neurotoxin [23] and sequence alignments revealed significant homologies with serotype families F and A. The LC protease subunit of BoNT/FA is 81% identical with BoNT/F5 (UniParc numbers: UPI10001C0B12E and UPI000521D14E), and the Hcc cell binding domain is 93% identical with BoNT/A1 (UniParc number: UPI0000001386) [20] and appears to share structural similarities with BoNT/A8 (UniParc number: UPI0003C9D2A1) [24]. Therefore, rather than being a new serotype, designation as a mosaic BoNT/FA seems more appropriate for this neurotoxin. Like other mosaic neurotoxin proteins, BoNT/FA contains regions that do not cluster clearly into any serotype phylogenetic group; the N-terminus of the heavy chain (Hn) ranges between 35% (with serotype C) and 61% (with serotype F) identity with other known serotypes [20].

The BoNT/FA LC protease domain is related functionally, as well as phylogenetically, to other F serotype family protease domains. It cleaves VAMP-2 between residues L54 and E55, which is the same site as BoNT/F5 (its closest homologue) but different from other BoNT/F serotype family members, which cleave VAMP-2 between residues Q58 and K59 [25,26,27,28,29]. There is also high sequence similarity between the cell binding regions of BoNT/FA and BoNT/A subtype proteins, which bind to the neuronal cell surface receptors glycosylated Synaptic Vesicle glycoproteins A, B and C (SV2A, B and C), and complex gangliosides such as GT1b and GD1a [13,30,31,32,33]. However, this relationship is more complex because structural analyses reveal differences that may explain differences in neutralization by antibodies and cell binding [24].

Generating sufficient purified protein for experiments is a necessary step to characterize any new BoNT and entails the choice of purifying either from a native *Clostridium botulinum* strain or from a heterologous expression system. There are strengths and weaknesses to these two approaches and it is attractive to do both. Pellett et al. have purified BoNT/FA from a genetically modified derivative of the native *C. botulinum* strain, in which they had selectively inactivated the gene encoding the BoNT/B serotype major toxin to allow purification of the BoNT/FA minor toxin [21,29]. Strengths of this approach are that the BoNT is expressed in an environment highly similar to the wild type and post-translational proteolytic activation is likely to occur by wild type proteases and/or in the presence of complexing proteins such as nontoxic, nonhemagglutinin (NTNHA) [34,35,36]. Weaknesses are that *C. botulinum* strains are not widely used as expression hosts in research laboratories. Very few laboratories have the capacity to handle neurotoxin expressing *C. botulinum* strains. They are complicated to culture and are spore-forming biohazardous microorganisms that require high levels of biocontainment. Purification of BoNT from *C. botulinum* is time consuming (11 days or more) [37] and can show significant batch to batch variation in the degree of post-translational modification and specific activity of the final product [38]. This leads to a requirement to measure the specific toxicity of every batch purified from native *Clostridium botulinum* in a mouse lethality assay to allow comparison based on equal lethal activity units rather than moles of protein. Also, other non-BoNT, *C. botulinum* proteins are poorly characterized. There is a risk that bioactive clostridial proteins, such as ADP-ribosylases, may copurify and affect the results of subsequent characterization experiments [39,40]. Here we report the alternative approach of over-expressing recombinant BoNT/FA in *Escherichia coli*. This complements the *C. botulinum* approach because *E. coli* is an established expression system, which is non-spore forming, has fast growth kinetics, well characterized host-cell proteins and well established methods for genetic modification of expressed proteins. Expression in *E. coli* is tractable, versatile and potentially achievable by many more research laboratories compared to expression in *C. botulinum*. Recombinant BoNTs purified from *E. coli* and post-translationally activated in in vitro (as an independently optimized step in the purification method) show consistent batch-to-batch specific activities and can be compared reliably based on equal moles of protein. However, the recombinant approach does have the weakness that BoNTs are expressed in a non-native environment. In particular, post-translational activation, albeit less variable, is by a non-natural route. The BoNT/FA proteins in this report also contain engineered modifications, such as affinity tags and changes to the activation loop, designed to facilitate purification and activation.

We have characterized recombinant BoNT/FA in a cell-free protease activity assay, two different primary neuron cell culture models for inhibition of neurotransmitter release and in an ex vivo mouse phrenic nerve hemidiaphragm assay for inhibition of neuromuscular signaling. The characteristics of the recombinant protein agree well with the native protein. We also purified enzymatically inactive BoNT/FA, containing inactivating amino acid substitutions within the catalytic site of the LC protease domain, as a source of nontoxic material suitable for biophysical characterization and generation of antibodies.

## 2. Results

### 2.1. Expression, Purification and Proteolytic Activation of Recombinant BoNT/FA

**Figure 1:**
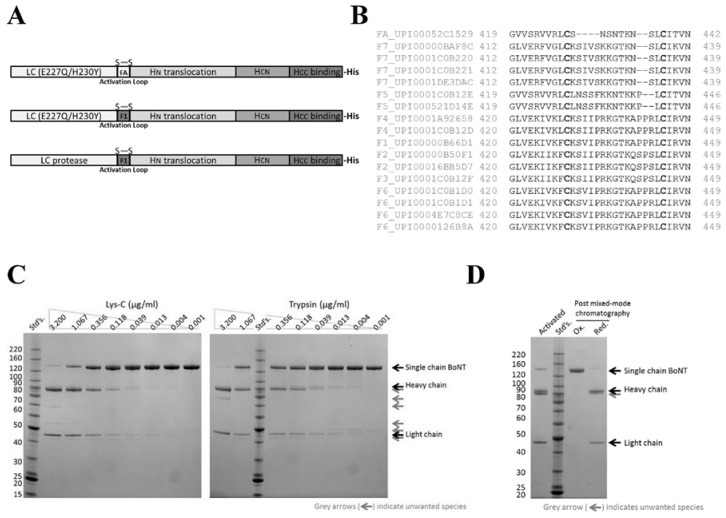
Purification of activated botulinum neurotoxin serotype FA (BoNT/FA). (**A**) Represents the domain structures of endopeptidase inactive rBoNT/FA(0)-his and mrBoNT/FA-his in which the activation loop has been replaced by that of BoNT/F1, and endopeptidase active mrBoNT/FA-his. (**B**) Shows a ClustalW alignment of BoNT/F subtype activation loops against BoNT/FA. The cysteine residues either side of the activation loops are shown in bold; amino acid positions within the full-length sequences and UniParc accession numbers are shown. (**C**) Shows SDS-PAGE analysis of activation products from 4 μM rBoNT/FA(0)-his incubated with 3.2–0.001 μg/mL Lys-C or trypsin, for 1 h at 37 °C. (**D**) Shows SDS-PAGE analysis of mrBoNT/FA(0)-his activated by Lys-C then further purified by mixed mode chromatography. The sample in the lane labeled Ox. was prepared in nonreducing SDS-PAGE sample buffer, samples in the other two lanes were further supplemented with 100 mM DTT.

The BoNT/FA gene sequence was codon optimized for expression in *E. coli* and used to construct three expression plasmids encoding: (1) rBoNT/FA(0)-his, which is recombinant BoNT/FA rendered endopeptidase inactive by two point mutations in the catalytic site (E227Q and H230Y) and carrying a C-terminal ten-histidine affinity tag (prefix r indicates recombinant, (0) indicates enzymatically inactive, suffix-his indicates ten-histidine affinity tag); (2) mrBoNT/FA(0)-his, which is endopeptidase inactive recombinant BoNT/FA in which the naturally occurring activation loop (amino acids S429 to L437 in UniParc sequence UPI00052C1529) is replaced by the activation loop from BoNT/F1 (K430 to L444 in UniParc sequence UPI00000B66D1) (prefix mr indicates modified recombinant); (3) mrBoNT/FA-his, which is endopeptidase active recombinant BoNT/FA containing the BoNT/F1 activation loop (Figure 1A and Supplemental Figures S1 and S2).

rBoNT/FA(0)-his, purified from *E. coli* by Ni^2+^ affinity chromatography, yielded between 3–10 mg/L of the single chain form. However, we were unable to identify suitable activation conditions to generate the mature heterodimeric form of this protein. A limited screen of proteolytic activation conditions by each of two different proteases did not identify suitable conditions for specific cleavage of the activation loop. The proteases were Endoproteinase Lys-C (Lys-C) and trypsin, tested between 3.2–0.001 μg/mL, for cleavage of 4 μM rBoNT/FA(0)-his, 1 h reaction time at 37 °C. Both proteases, in our hands, cleaved additional sites outside of the activation loop and created unwanted truncated species visible by SDS-PAGE (Figure 1).

Sequence alignment of the activation loop from BoNT/FA with those from other F serotype family members showed variability across this region (Figure 1B). The activation loop of BoNT/FA is six amino acids shorter compared to other family members and we speculated that the short loop of BoNT/FA might be less accessible to proteases. We had previously identified conditions under which the activation loop of rBoNT/F1 is cleaved selectively by Lys-C (data not shown) and hypothesized that this activation loop might retain sensitivity to selective activation within the context of the BoNT/FA protein. mrBoNT/FA(0)-his is a modified BoNT/FA containing the activation loop from BoNT/F1 (K430-L444). This protein was expressed and purified with similar yields to rBoNT/FA(0)-his. Lys-C did indeed cleave more selectively within the longer activation loop. Unwanted truncated species were generated to a lesser extent and we were able to purify activated heterodimeric mrBoNT/FA(0)-his by a subsequent step of mixed-mode chromatography (Figure 1D and Methods Section). Three independent batches of the endopeptidase active form, mrBoNT/FA-his, purified by the same method under biosafety level 2 plus containment, yielded between 0.1–0.7 mg/L *E. coli* culture.

**Figure 2:**
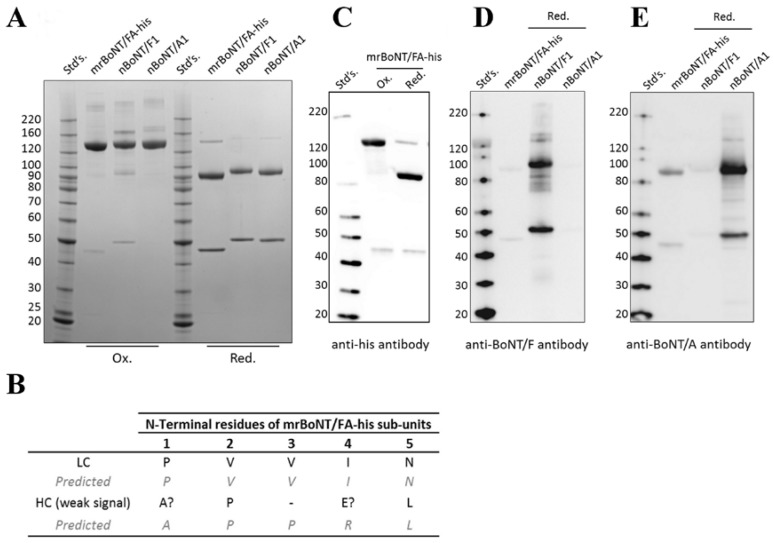
Protein analysis of mrBoNT/FA. (**A**) Shows SDS-PAGE analysis of mrBoNT/FA-his compared to nBoNT/F1 (Metabiologics) and nBoNT/A1 (Metabiologics,). Samples in the lanes labeled Ox. were prepared in nonreducing SDS-PAGE sample buffer, samples in lanes labeled Red were further supplemented with 100 mM DTT. (**B**) Shows results from N-terminal sequencing of mrBoNT/FA-his LC and HC chain species excised from SDS-PAGE gels. Letters correspond to the single character amino acid code for the detected (black) and predicted (grey) amino acid at each location, a “?” symbol indicates inconclusive data, and a “-“ symbol indicates no data for that position. (**C**) Shows Western blot analysis of mrBoNT/FA detected by an anti-his tag primary antibody. (**D**) Shows Western blot analysis of mrBoNT/FA, nBoNT/F1, and nBoNT/A1 detected by an anti-BoNT/F primary antibody. (**E**) s\Shows Western blot analysis of mrBoNT/FA, nBoNT/F1 and nBoNT/A1 detected by an anti-BoNT/A primary antibody.

**Figure 3:**
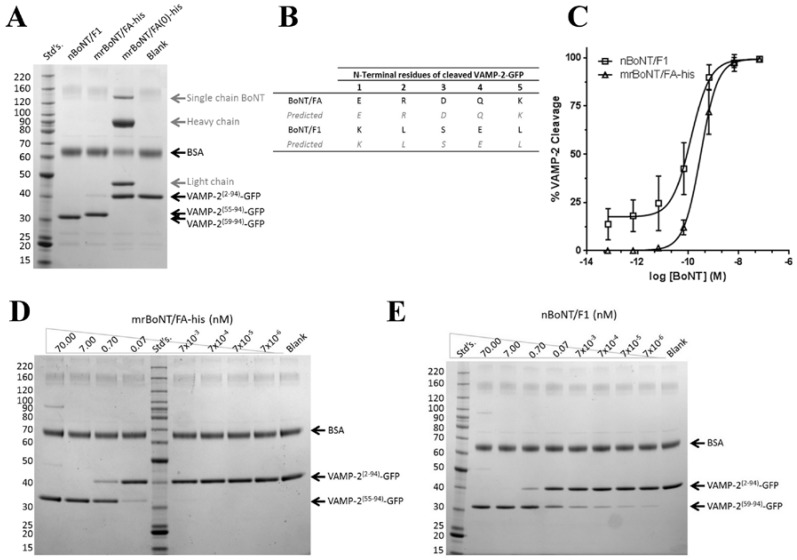
Cell free proteolysis of recombinant vesicle associated membrane protein-2 (VAMP-2). (**A**) Shows SDS-PAGE analysis of cell-free proteolysis of a recombinant VAMP-2 substrate (VAMP-2^(2–94)^-GFP) by nBoNT/F1 (7 nM), mrBoNT/FA-his (7 nM) and mrBoNT/FA(0)-his (2 μM). (**B**) Shows results from N-terminal sequencing of the cleaved products excised from SDS-PAGE gels. Letters correspond to the single character amino acid code for the detected (black) and predicted (grey) amino acid at each location. (**C**) Shows quantification of VAMP-2^(2–94)^-GFP cleavage, determined by densitometry of SDS-PAGE gels stained for total protein, by nBoNT/F1 and mrBoNT/FA-his. Data were fitted to a four-parameter equation and represent means ± s.e.m. of *n* = 4 independent experiments. (**D**,**E**) Show SDS-PAGE analyses of cell-free VAMP-2^(2–94)^-GFP proteolysis by various concentrations of mrBoNT/FA-his and nBoNT/F1 respectively.

The calculated molecular weights, based on the primary sequence, of the BoNT/FA LC and HC subunits are 48.8 kDa and 100.7 kDa, respectively. The purified recombinant proteins migrated with lower than expected apparent molecular weights on SDS-PAGE (Figure 2 and Figure 3). However, a combination of N-terminal sequencing, Western blot data, and the effects of reducing agents suggest that both subunits have intact N- and C-termini and are linked by an interchain disulphide bond. N-terminal sequencing is consistent with the expected N-terminal residues for the LC and HC subunits (Figure 2B). Western blotting for the C-terminal histidine tag detected immunoreactive species consistent with the HC subunit having an intact C-terminus (Figure 2C). Under nonreducing conditions, mrBoNT/FA(0)-his migrated as a single species on SDS-PAGE (apparent molecular weight approximately 150 kDa). Under reducing conditions, it migrated as two species (approximately 50 and 100 kDa) (Figure 2). This pattern is consistent with the presence of an intact disulphide bridge between C-terminal LC and N-terminal HC cysteine residues [41]. The HC subunit was also immunoreactive to a polyclonal anti-BoNT/A (Metabiologics, Madison, WI, USA) on Western blots (Figure 2E). This is consistent with the high sequence homology with the HC of BoNT/A1. In contrast, a polyclonal anti-BoNT/F1 (Abcam, Cambridge, UK, Ab27168) did not detect BoNT/FA (Figure 2D). LC/FA is 50% identical with BoNT/F1, against which this antiserum was raised; one possibility is that the LC/FA may not contain the LC/F1 epitopes to which this antiserum binds.

### 2.2. VAMP-2 Cleavage by BoNT/FA

Both mrBoNT/FA-his and nBoNT/F1 (Metabiologics) (prefix n indicates native toxin, purified from *C. botulinum*) cleaved a recombinant VAMP-2 substrate (VAMP-2^(2–94)^-GFP) in cell-free proteolysis assays [42]; 7 nM mrBoNT/FA-his and 0.7 nM nBoNT/F1 were sufficient to cleave nearly 100% of 4 μM VAMP-2^(2–94)^-GFP in one hour at 37 °C (Figure 3). In contrast, 2 μM of the endopeptidase inactive mrBoNT/FA(0)-his did not cleave any detectible VAMP-2^(2–94)^-GFP under the same conditions (Figure 3A). N-terminal sequencing of the cleaved products from VAMP-2^(2–94)^-GFP confirmed that mrBoNT/FA-his cleaved at the BoNT/F5 site, between residues L54 and E55, and nBoNT/F1 cleaved at the BoNT/F1 site, between residues Q58 and K59 (Figure 3B). mrBoNT/FA-his VAMP-2^(2–94)^-GFP cleavage activity fit well to a four-parameter equation with parameter values of: bottom asymptote 0.2%, top asymptote 99.4%, midpoint −9.503 log M (0.3 nM), and Hill slope 1.3 (*R*^2^ = 0.991). In contrast, the nBoNT/F1 activity did not fit to four-parameter equations where the bottom asymptote approached zero cleavage at enzyme concentrations approaching zero. The best-fit parameter values were for a bottom asymptote of 17.5%, with top asymptote 99.3%, midpoint −9.884 log M (0.1 nM), and Hill slope 1.1 (*R*^2^ = 0.950) (Figure 3C). mrBoNT/FA-his showed lower specific activity than nBoNT/F1. Whereas mrBoNT/FA-his caused no detectable VAMP-2^(2–94)^-GFP cleavage in this assay at concentrations below 7 × 10^−3^ nM (Figure 3D), nBoNT/F1 concentrations as low as 7 × 10^−6^ nM were sufficient to generate detectable cleaved product (Figure 3E).

### 2.3. Inhibition of Neurotransmitter Release in Rat Embryonic Spinal Cord Neurons and Rat Cortical Neurons by BoNT/FA

**Figure 4:**
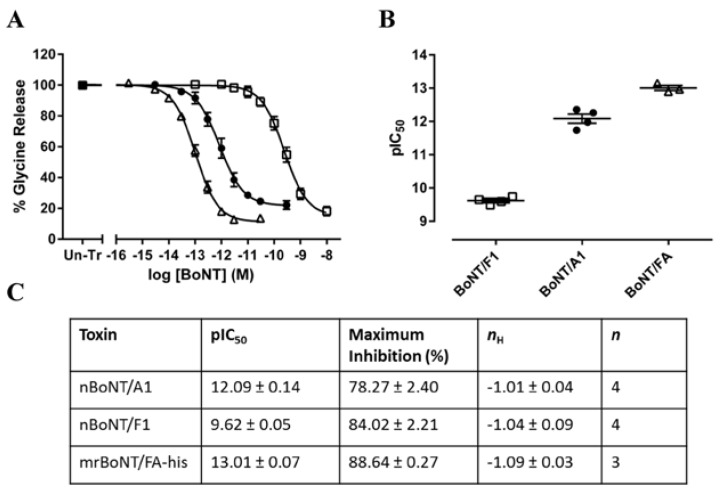
Inhibition of [^3^H]-glycine release from rat embryonic spinal cord neurons. (**A**) Shows data from rat embryonic spinal cord neurons treated with nBoNT/F1 (□), nBoNT/A1 (●) or mrBoNT/FA-his (△) for 24 h. Cells were loaded with [^3^H]-glycine for 60 min in HEPES Buffered Salt solution (HBS) before washing and assessment of basal and stimulated [^3^H]-glycine release from 5-min exposure to HBS supplemented with 3 mM or 60 mM potassium, respectively. Radioactivity associated with cell superfusates was determined by liquid scintillation counting. Data were normalized to the [^3^H]-glycine released by un-treated cells (Un-Tr) and fitted to a four-parameter equation. (**B**) Shows the concentration of each toxin required for 50% maximal inhibition (pIC_50_) of [^3^H]-glycine release, determined from the fitted curves. The table in (**C**) shows the pIC_50_, maximum inhibition, Hill slope (nH), and number of independent experiments; data are means ± s.e.m.

**Figure 5:**
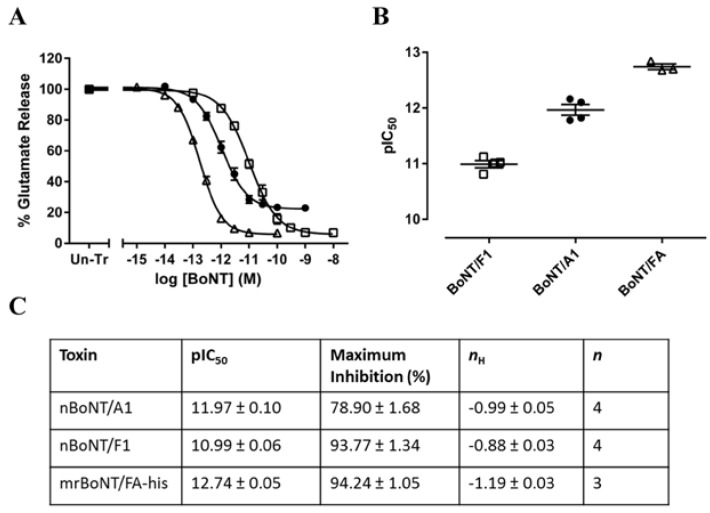
Inhibition of glutamate release from rat cortical neurons. (**A**) Shows data from rat cortical neurons treated with nBoNT/F1 (□), nBoNT/A1 (●) or mrBoNT/FA-his (△) for 24 h. Cells were preincubated for 30 min in assay buffer containing the excitatory amino acid transporter inhibitor (3S)-3-[[3-[[4-(Trifluoromethyl)benzoyl]amino]phenyl]methoxy]-l-aspartic acid (TFB-TBOA) (10 μM) then assessed for basal and stimulated glutamate release after 5 min exposure to assay media containing 5 mM or 60 mM potassium, respectively. Glutamate content of cell superfusates was measured by an Amplex Red fluorometric assay. Data were normalized to the glutamate release from untreated cells (Un-Tr) and fitted to a four-parameter equation. (**B**) Shows the concentration of each toxin required for 50% maximal inhibition (pIC_50_) of glutamate release, determined from the fitted curves. The table in (**C**) shows the pIC_50_, maximum inhibition, Hill slope (nH), and number of independent experiments; data are means ± s.e.m.

mrBoNT/FA-his, nBoNT/F1 and nBoNT/A1 inhibited neurotransmitter release from intoxicated primary cultures of rat neurons. These BoNTs all caused concentration dependent inhibition of pre-loaded [^3^H]-glycine release from rat embryonic spinal cord neurons and of endogenous glutamate release from rat cortical neurons. In rat embryonic spinal cord neurons, mrBoNT/FA-his was significantly more potent (pIC_50_ = 12.90 ± 0.17) than either nBoNT/A1 (pIC_50_ = 12.09 ± 0.14) or nBoNT/F1 (pIC_50_ = 9.62 ± 0.05) (Figure 4). Similarly, in rat cortical neurons, mrBoNT/FA-his was more potent (pIC_50_ = 12.70 ± 0.07) than either nBoNT/A1 (pIC_50_ = 11.97 ± 0.10) or nBoNT/F1 (pIC_50_ = 10.99 ± 0.06) (Figure 5).

### 2.4. SNARE Cleavage in Rat Cortical Neurons by BoNT/FA

**Figure 6:**
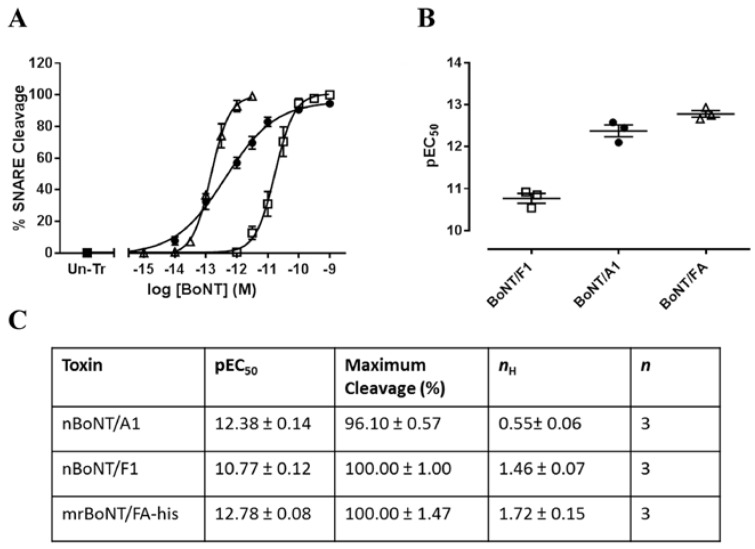
SNARE cleavage in rat cortical neurons. (**A**) shows data from rat cortical neurons treated with nBoNT/F1 (□), nBoNT/A1 (●) or mrBoNT/FA-his (△) for 24 h. Cell were lysed, run on SDS-PAGE and blotted for VAMP-2 or SNAP-25. The percentage of SNARE cleaved was determined from the ratio of full length to cleaved protein by densitometric analysis. Data were fitted to a four-parameter equation. (**B**) shows the concentration of each toxin required for 50% maximal SNARE cleavage (pEC_50_), determined from the fitted curves. The table in (**C**) shows the pEC_50_ and number of independent experiments; data are means ± s.e.m.

mrBoNT/FA-his, nBoNT/F1 and nBoNT/A1 cleaved SNARE proteins in intoxicated rat cortical neurons. Cells tested in glutamate release assays were subsequently lysed and Western blotted for VAMP-2 (nBoNT/F1 and mrBoNT/FA-his) or SNAP-25 (nBoNT/A1) (Supplemental Figure S3). mrBoNT/FA-his cleaved VAMP-2 with potency pEC_50_ = 12.75 ± 0.14. nBoNT/F1 cleaved VAMP-2 with potency pEC_50_ = 10.77 ± 0.12. nBoNT/A1 cleaved SNAP-25 with potency pEC_50_ = 12.38 ± 0.14, *n* = 3 (Figure 6).

### 2.5. Paralysis of Mouse Hemi-Diaphragm Contraction by BoNT/FA

**Figure 7:**
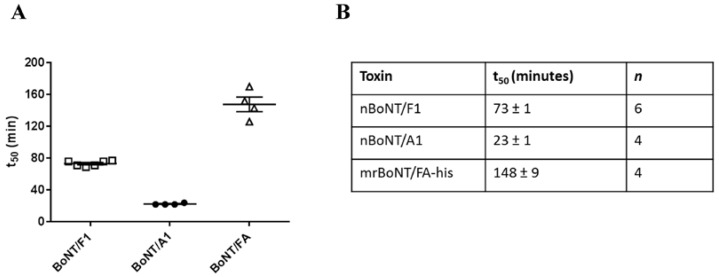
Inhibition of neuromuscular signaling in mouse phrenic nerve hemidiaphragm assays. (**A**) shows the time taken for 100 pM nBoNT/F1(□), nBoNT/A1 (●), or mrBoNT/FA-his (△) to reduce the force of contraction to 50% of the starting value (t_50_) in ex vivo mouse phrenic nerve hemidiaphragm preparations. The table in (**B**) shows the mean t_50_ values ± s.e.m and the number of independent experiments.

mrBoNT/FA-his, nBoNT/F1 and nBoNT/A1 inhibited nerve-stimulated muscle contraction in the mouse phrenic nerve hemidiaphragm (mPNHD) assay; 100 pM mrBoNT/FA-his, nBoNT/A1 or nBoNT/F1 reduced the force of contraction to 50% of the starting value with half-times (t_50_) of: 148 ± 9 min (mrBoNT/FA-his), 73 ± 1 min (nBoNT/F1), and 23 ± 1 min (nBoNT/A1). mrBoNT/FA-his was significantly less potent than either nBoNT/A1 or nBoNT/F1 in this assay (Figure 7).

## 3. Discussion

The mosaic structure of BoNT/FA combines an F5 family LC protease subunit with an A1 family Hcc cell-binding domain and may confer unanticipated and potentially therapeutically valuable properties to this neurotoxin. Other naturally occurring mosaic BoNTs, such as BoNT/DC, have characteristics that are different from the constituent serotypes [43,44]. Similarly, engineered recombinant mosaic toxins that combine domains from BoNT/A with BoNT/E or BoNT/B have characteristics such as potency, toxicity and duration of action not predicted from those of their constituent serotypes [45,46]. Therefore, we set out to develop a tractable system to express, purify, characterize, and modify recombinant BoNT/FA.

BoNT expression in *E. coli* offers several advantages over *C. botulinum*. These include rapid and reproducible growth of expression cultures, established genetic methods to create point mutations, deletions, and add purification tags, and a well-characterized expression background that makes it easier to identify and remove unwanted copurifying proteins. We exploited these properties to construct and purify enzymatically active and inactive variants of BoNT/FA. The enzymatically inactive variants contained inactivating point mutations (E227Q and H230Y) in the catalytic site that disrupt a critical HExxH motif of the protease and abolish Zn^2+^ binding [47,48,49]. Because protease activity is essential for the neurotoxic activity, such mutations render the protein nontoxic [49]. The availability of nontoxic BoNT/FA(0) is important because it will allow further biophysical characterization of the protein, optimization of conditions for post-translational modification (to increase yields), optimization of classical purification methods (to produce untagged BoNT/FA), generation of specific antibodies, and provide material that can be handled safely in large quantities for structural studies such as X-ray crystallography [50].

Wild type and recombinant BoNT proteins are expressed as single chain protoxins that become post-translationally modified by proteolytic cleavage to generate fully-active, mature forms [21,51,52,53,54]. Non-native expression systems, such as *E. coli*, as well as several strains of *C. botulinum* (called nonproteolytic strains) do not express an endogenous protease that can generate the mature form within the expression culture. Rather, the single chain protein becomes further processed by a suitable exogenous protease. The naturally occurring protease or proteases that activate BoNT proteins in the wild are poorly characterized, but exogenous proteases, such as trypsin, are commonly used to activate BoNTs purified from nonproteolytic *C. botulinum* strains [21,54,55,56,57]. Indeed, native BoNT/FA bound to NTNHA in extracts from the CDC69016/B2tox^−^ *C. botulinum* strain can be activated by trypsin [21]. However, binding to NTNHA protects BoNT proteins from proteolysis. Purified, noncomplexed, BoNT proteins are more susceptible [35]. Suitable proteases and the reaction conditions to achieve specific proteolysis of purified 150 kDa BoNT proteins are different for individual BoNTs and must be determined empirically. For example, trypsin is not suitable for activation of purified single chain nBoNT/A1 because it also cleaves nBoNT/A1 outside of the activation loop, within the HC domain, during post-translational activation reactions [35,58]. Here, we screened a range of concentrations of trypsin and of Endoproteinase Lys-C searching for suitable activation conditions. However, none of the tested conditions efficiently cleaved mrBoNT/FA(0)-his within the activation loop (between C428 and C444) without also cleaving at other unwanted sites in the protein. Our screening results suggest that purified BoNT/FA is like BoNT/A in that it is susceptible to unwanted cleavage events. The naturally occurring activation loop region of BoNT/FA is shorter than that of other BoNT/F family members. In particular, it is shorter than the activation loop of BoNT/F1 and we had previously identified in vitro reaction conditions under which Lys-C cleaves specifically inside the activation loop of BoNT/F1. We reasoned that the longer activation loop of BoNT/F1 might be more accessible to Lys-C and might retain this accessibility within the context of BoNT/FA. Therefore, we substituted the BoNT/FA activation loop (S429-L437) for that of BoNT/F1 (K430-L444). This substitution did indeed allow us to identify conditions under which Lys-C cleaved within the activation loop more efficiently. We identified conditions under which approximately 50% of the protein appeared to be the nontruncated mature form and a subsequent step of mixed-mode chromatography was sufficient to separate this material. Based on this observation, we speculate that the activation loop length is an important factor in post-translational proteolytic activation to generate mature form di-chain toxins. Since the activation loops of BoNTs are poorly conserved and lie outside of structural domains, we believe that substituting the activation loop in this way is unlikely to have changed the characteristics of the activated protein. Indeed, another strategy commonly employed by researchers in the field is to substitute wild type activation loop sequences of recombinant BoNT proteins with consensus recognition motifs of proteases such as thrombin [59].

The specific activity of mrBoNT/FA-his was significantly lower than nBoNT/F1 in cell-free protease assays. N-terminal sequencing of the cleaved product confirmed that the cleaved peptide bond in human VAMP-2 was between residues L54 and E55, which is consistent with the sequence based identification of the BoNT/FA protease subunit as a BoNT/F5 family member, and with previous reports [20,26]. The different site of cleavage in VAMP-2 between BoNT/FA and BoNT/F1 is also evident from the different sized cleavage products in cell-free and cell assays (Figure 3 and Supplementary Figure S3). The high sequence homology between the BoNT/FA and BoNT/F5 protease domains, together with the shared cleavage site, suggests that BoNT/FA most likely binds and cleaves VAMP-2 by the same mechanism as reported for BoNT/F5 [27,60]. Further studies to elucidate the properties of BoNT/F5 and BoNT/FA substrate binding and cleavage will help to inform the design of novel small molecule inhibitors directed towards these two BoNTs. We also note the striking difference in shape of the dose-response curves for substrate cleavage by BoNT/FA and BoNT/F1. Further investigation into the enzyme kinetics of these two proteases may be insightful.

We compared inhibition of neurotransmitter release in two primary neuron cell models by mrBoNT/FA-his, nBoNT/F1, and nBoNT/A1. The models were cultured rat embryonic spinal cord neurons and rat cortical neurons. The rank order of potency was the same in both cell types BoNT/FA > A1 > F1 but the magnitude of difference varied. mrBoNT/FA-his was approximately one log unit more potent than nBoNT/A1 in both cell types. However, the size of the difference between mrBoNT/FA-his and nBoNT/F1 was different between these two cell types. mrBoNT/FA-his was approximately three log units more potent than nBoNT/F1 in rat embryonic spinal cord neurons compared to approximately one log unit in cortical neurons. This difference was driven by the relatively low potency of nBoNT/F1, especially in rat embryonic spinal cord neurons (pIC_50_ 9.62 for BoNT/F1, compared to 12.09 and 12.90 for nBoNT/A1 and rBoNT/FA, respectively). The structure of the BoNT/FA cell-binding domain suggests it may interact differently with neuronal cell surface receptors compared to BoNT/A1 [24,33]. BoNT/F1 also may interact differently with cell surface receptors compared to both BoNT/FA and BoNT/A. Competition assays between the HC domains of BoNT/F, BoNT/A and BoNT/E suggest that BoNT/F1 interacts differently with SV2 compared to BoNT/A1 [61,62]. Therefore, the large difference in relative potency between BoNT/FA and BoNT/F1 in intoxicating rat embryonic spinal cord neurons may reflect different receptor populations between these cell types.

The high potency with which mrBoNT/FA-his inhibits neurotransmitter release in primary neurons was not reflected in the ex vivo mouse phrenic nerve hemidiaphragm (mPNHD) assay, which measures nerve-stimulated skeletal muscle contraction. In this assay, BoNT/FA was significantly less potent than either nBoNT/A1 or nBoNT/F1. This was not due to a species difference because we also saw the same pattern in ex vivo rat phrenic nerve hemidiaphragm preparations (data not shown), nor to different expression levels of VAMP isoforms 1 and 2 between peripheral and central neurons because BoNT/FA cleaves both with similar efficiency [29]. One important difference between the mPNHD assays and cell assays is the shorter assay time between exposure to BoNT and measurement of effect. We measured the effects of intoxication 24 h after exposure of cultured neurons. In contrast, effects on muscle contraction in the mPNHD assay were in real time, starting immediately after exposing the tissue to the toxin, and for a maximum of 4 h. One possibility is that the slower enzyme kinetics of the BoNT/FA protease may cause the low potency inhibition of muscle contraction in the mPNHD assay. Other possible explanations include that intoxication of peripheral motor neurons may be different from intoxication of central spinal cord or cerebellar neurons; or that the tissue may contain additional cell types or molecules, which are not present in cultures of primary neurons, and which reduce the potency of BoNT/FA. The difference between activity in cell assays and in mPNHD tissue is consistent with observations reported by Pellett et al., that BoNT/FA purified from a modified CDC69016 strain of *C. botulinum* shows higher activity compared to BoNT/A1 in cultured neurons but lower activity in a mouse bioassay [21,29].

The approach we have taken to purifying BoNT/FA is a tractable method to generate highly pure homogeneous preparations of enzymatically active, and of enzymatically inactive, material suitable for biological, biophysical, and structural characterization. Such studies are essential to achieve further insights into the biological properties of different BoNT protein families and to realize the potential to exploit those differences to develop novel BoNTs with unique therapeutic properties.

## 4. Materials and Methods

### 4.1. Materials 

Oligonucleotides were synthesized by Eurofins Genomics, Ebersberg, Germany. The expression plasmid was pJ401 from DNA2.0. All other reagents were from Sigma-Aldrich now Merck, Gillingham, Dorset UK, unless otherwise stated. All protein expression was performed by transforming chemically competent BL21 (DE3) *E. coli* (Novagen, Birmingham, UK) with appropriate pJ401 expression plasmids.

### 4.2. Molecular Cloning

The codon optimized, synthetic gene encoding the BoNT/FA protein (UniParc: UPI00052C1529) with a BoNT/F1 activation loop (UniParc: UPI00000B66D1) and a C-terminal ten-histidine tag (-his) was synthesized and cloned into the pJ401 vector (DNA2.0). The BoNT/FA nucleotide sequence, possessing the native loop and the mutations E227Q and H230Y, were generated by site-directed mutagenesis of the synthetic BoNT/FA sequence using the quick-change method and a KOD Hot Start DNA Polymerase (Merck Millipore, Burlington, MA, USA). The mutagenic primer sequences were: AF(0)For CTTATGCATCAGTTGATTTACGTTTTGCATGG, AF(0)Rev CCATGCAAAACGTAAATCAACTGATGCATAAG, AF-loop-For GAGTCGCGTCGTTCGCTTGTGCAGTAATAGTAATACCAAGAATAGTCTGTGTATCACCGTGAATAACC, AF-loop-Rev GGTTATTCACGGTGATACACAGACTATTCTTGGTATTACTATTACTGCACAAGCGAACGACGCGACTC. All nucleotide sequences were confirmed by Sanger sequencing.

### 4.3. Protein Expression and Purification

Neurotoxins were expressed, purified, and handled in microbiological safety cabinets Type I (MSCI) in a containment biosafety level 2 laboratory. All proteins were expressed in the BL21 (DE3) strain of *E. coli* transformed with the pJ401-BoNT expression plasmid under induction by 1 mM Isopropyl β-ᴅ-1-thiogalactopyranoside (IPTG) for 20 h at 16 °C. All expression cultures were prepared at a 1 L scale in sterilized modified Terrific Broth (mTB) (Melford Biolaboratories Ltd., Suffolk, UK) containing 0.2% glucosamine and 30 μg/mL kanamycin. Cell pellets were lysed by sonication in lysis buffer (50 mM Bis-Tris propane, pH 7.0, 500 mM NaCl), supplemented with Benzonase^®^ Nuclease (1:6000) (Sigma-Aldrich now Merck Gillingham, Dorset, UK) and subsequently clarified by centrifugation. Clarified lysate was loaded onto a charged Ni^2+^ Chelating column (HisTrap HP, GE Healthcare, Little Charpentine, UK) on an ÄKTA purifier (GE Healthcare). The column was washed with 50 mM Bis-Tris propane (pH 7.0) 50 mM NaCl, 80 mM Imidazole. The remaining bound protein was eluted with 50 mM Bis-Tris propane (pH 7.0) 50 mM NaCl, 250 mM Imidazole. Eluted BoNT/FA was buffer exchanged into activation buffer (50 mM Bis-Tris propane (pH 7.0) 125 mM NaCl) by diafiltration. The single chain BoNT/FA was diluted to 0.68 mg/mL and treated with 0.05 μg/mL Endoproteinase Lys-C (Sigma-Aldrich) at 37 °C for 2 h. The activated sample was then buffer exchanged into 25 mM Sodium Phosphate buffer (pH 6.5), loaded onto a CHT type II (Bio-Rad, Hercules, CA, USA) column, and eluted over a phosphate buffer gradient of 25–500 mM Sodium Phosphate buffer (pH 6.5). The eluted BoNT/FA was buffer exchanged into Gibco^®^ PBS (pH 7.2), diluted to 0.1 mg/mL and supplemented with 1 mg/mL BSA.

### 4.4. SDS-PAGE and Western Blot Analysis

Protein samples were loaded onto NuPAGE 4–12% Bis-Glycine Gels (1 μg per lane; 1.0 mm, 10 wells) and run at 200 V for 50 min. Gels were stained with Simply Blue SafeStain (ThermoFisher Scientific, Waltham, MA, USA) and imaged using a Syngene PXi instrument. Alternatively, proteins were transferred to nitrocellulose membranes for Western blot. Antibodies for BoNT/A domains were primary: goat anti-BoNT/A IgG (Metabiologics, 0.24 μg/mL) and secondary: horseradish peroxidase (HRP)-conjugated anti-goat IgG (Sigma-Aldrich). Antibodies used for BoNT/F domains were primary: rabbit anti-BoNT/F1 pAb (Abcam ab27168, 1/5000) and secondary: HRP-conjugated anti-rabbit IgG (Sigma-Aldrich). Blots were developed with SuperSignal West Dura Chemiluminescent Substrate and visualized using a Syngene PXi instrument.

### 4.5. Cell-Free VAMP-2 Proteolysis Assay

mrBoNT/FA-his and nBoNT/F1 (Metabiologics) were diluted to 0.2 mg/mL and titrated (10-fold serial dilutions) in assay buffer (50 mM HEPES, pH 7.2, 20 μM ZnCl2, 1 μg/μL BSA, 10 mM DTT). The soluble region of human VAMP-2 (amino acids 2–94) was expressed in *E. coli* with a cleavable N-terminal GST and a C-terminal GFP tag. The protein was purified on glutathione sepharose resin (GE Healthcare Life Sciences), the GST tag cleaved (PreScission protease), then both the cleaved GST-tag and the PreScission protease (itself GST-tagged) were cleared by a second pass over glutathione sepharose resin. Each diluted BoNT was mixed with an equal volume of 8 μM recombinant VAMP-2^(2–94)^-GFP solution in assay buffer and incubated at 37 °C for 1 h. Reactions were terminated by addition of Novex 2× LDS sample buffer (ThermoFisher Scientific) and heating at 95 °C for 5 min. Samples were loaded onto 4–12% Bis–Tris gels (Thermo Fisher Scientific) alongside BenchMark molecular weight markers (Thermo Fisher Scientific), and visualized by staining with Simply Blue Safestain (Thermo Fisher Scientific). Assay results were quantified by densitometry (Syngene Bioimaging) and the proportion of cleaved VAMP-2^(2–94)^-GFP (% cleaved) was plotted against the BoNT concentration. VAMP-2 cleaved products (approximately 31–32 kDa) were excised from the SDS-PAGE gel and analyzed by Edman degradation (Alta Bioscience, Edgbaston, UK) to determine the site of cleavage. The effect of point mutations E227Q/H230Y in mrBoNT/FA(0)-his was evaluated by incubating 2 μM mrBoNT/FA(0)-his with 4 μM VAMP-2^(2–94)^-GFP at 37 °C for 1 h then stopping and analyzing the reaction as above.

### 4.6. Rat Cortical Neuronal Cell Culture

Rat cortical neurons were prepared from embryonic Day 17 to 18 (E17–E18) Sprague Dawley rat embryos. Dissected cortical tissue was collected into ice-cold Hank’s Balanced Salt Solution (HBSS), without Ca^2+^ or Mg^2+^, and then dissociated in papain solution for 40 min at 37 °C following the manufacturer’s instructions (Worthington Biochemical, Lakewood, NJ, USA). Cortical cells were plated on poly-l-ornithine (PLO) coated 96-well plates at a density of 20,000 cells/well in 125 μL Neurobasal media containing 2% B27 supplement, 0.5 mM GlutaMAX, 1% fetal bovine serum (FBS) and 100 U/mL penicillin/streptomycin. Cells were maintained at 37 °C in a humidified atmosphere containing 5% CO_2_. A further 125 μL Neurobasal media containing 2% B27, 0.5 mM GlutaMAX was added on the 4th day in vitro (DIV 4). Cells were maintained by replacement of half media twice per week. On DIV 11, 1.5 μM cytosine β-d-arabinofuranoside (AraC) was added to the media to prevent proliferation of non-neuronal cells.

### 4.7. Assessment of Glutamate Release from Rat Cortical Neurons Treated with Botulinum Neurotoxin

Glutamate release was assessed in cortical neurons at DIV 19–21. Cortical neurons were treated with a concentration-range of nBoNT/A (LIST Laboratories, Campbell, CA, USA), nBoNT/F1 (Metabiologics), or mrBoNT/FA-his for 24 h at 37 °C. Following removal of neurotoxin, cells were briefly washed 3 times in Neurobasal media containing 2% B27, 0.5 mM GlutaMAX and then pre-incubated in assay media (Neurobasal w/o phenol red, 2% B27, 0.5 mM GlutaMAX, 10 μM (3S)-3-[[3-[[4-(Trifluoromethyl)benzoyl]amino]phenyl]methoxy]-l-aspartic acid (TFB-TBOA) (excitatory amino acid transporter inhibitor, Tocris, Bristol, UK)) on a heat block at 35 °C for 30 min. Following pre-incubation, cells were briefly washed once in assay media. Basal and stimulated glutamate release were established by incubation at 35 °C for 5 min with 40 μL/well assay media containing low potassium (5 mM KCl), or high potassium (60 mM KCl), respectively. Cell superfusates were collected and glutamate content assessed using an Amplex Red glutamic acid assay (Invitrogen, Carlsbad, CA, USA). 10 μL superfusates were combined with 10 μL detection mix (100 mM Tris-HCl, pH 7.4 containing 26 μg/mL Amplex UltraRed, 0.25 U/mL horseradish peroxidase, 0.08 U/mL glutamate oxidase, 0.5 U/mL glutamate pyruvate transaminase and 200 μM alanine) in black 384-well Optiplates (Perkin Elmer, Waltham, MA, USA). Plates were incubated for 30 min at 37 °C after which 5 μL Amplex Red Stop reagent was added to each well. Fluorescence emission at 590 nm following excitation at 535 nm was determined using an Envision plate reader (Perkin Elmer). Glutamate concentrations of superfusates were determined by interpolation from a glutamate standard curve also run in each assay.

### 4.8. Rat Embryonic Spinal Cord Neuronal Cell Culture

Rat embryonic spinal cord neurons were prepared from E15 Sprague-Dawley rat embryos as described previously [63]. Briefly, dissected spinal cords were dissociated in trypsin-EDTA for 45 min at 37 °C, then triturated to a single cell suspension in plating media (minimal essential medium, (MEM) containing 2 mM GlutaMAX, 5% horse serum, 0.6% d-glucose and 0.15% NaHCO3). Cells were plated in matrigel coated 96-well plates at a density of 125,000 cells/well in 125 μL plating media. Cells were maintained at 37 °C in a humidified atmosphere containing 10% CO_2_. 24 h after plating, media was replaced with 125 μL MEM containing 2 mM GlutaMAX, 5% horse serum, 0.6% d-glucose, 0.15% NaHCO_3_, 2% N_2_ supplement, 40 ng/mL corticosterone, 20 ng/mL triiodothyronine. On DIV 6, 60 μM 5-fluoro-2′-deoxyuridine (FdU) and 14 μM uridine (U) were added to the media to prevent proliferation of non-neuronal cells. The media in each well was doubled to 250 μL on DIV 8. Cells were then maintained by replacement of half media twice per week.

### 4.9. Assessment of Glycine Release from Rat Spinal Cord Neurons Treated with Botulinum Neurotoxin

Glycine release was assessed in spinal cord neurons at DIV 20–23. Spinal cord neurons were treated with a concentration range of BoNT/A (List Biological Laboratories, Campbell, CA, USA), BoNT/F1 (Metabiologics), or BoNT/FA (30 pM–300 aM) for 24 h at 37 °C. Following removal of neurotoxin, cells were briefly washed 3 times in HEPES-buffered salt solution (HBS) (136 mM NaCl, 3 mM KCl, 2 mM CaCl_2_, 1 mM MgCl_2_, 10 mM HEPES, 10 mM glucose, pH 7.2). Cells were loaded with 2 μCi/mL [^3^H]-glycine (Perkin Elmer) in HBS for 60 min at 35 °C. Following removal of [^3^H]-glycine, cells were briefly washed 3 times with HBS. Basal and stimulated [^3^H]-glycine release were established by incubation at 35 °C for 5 min with 50 μL/well assay media containing low potassium (3 mM KCl), or high potassium (60 mM KCl) HBS solutions, respectively. To determine retained [^3^H]-glycine, cells were lysed by adding 50 μL/well RIPA buffer (Sigma-Aldrich). Superfusates and cell lysates were transferred into 96-well Isoplates (Perkin Elmer) and 200 μL/well OptiPhase Supermix scintillation fluid was added. Radioactivity was quantified using a MicroBeta2 plate reader (Perkin Elmer).

### 4.10. SDS-PAGE and Western Blot of Rat Cortical Neurons

After assessment of glutamate release, rat cortical neurons were lysed in 40 μL of lysis buffer (NuPage LDS sample buffer, 1 mM DTT and 1:500 Benzonase) for 10 min at room temperature. Samples were boiled at 90 °C for 5 min and 15 μL lysates loaded per lane to 12% Bis-Tris gels and run in either 3-(*N*-morpholino)propanesulfonic acid (MOPS) buffer at 200 V for 80 min (SNAP-25) or 2-(*N*-morpholino)ethanesulfonic acid (MES) buffer at 200 V for 50 min (VAMP-2). Proteins were transferred to nitrocellulose membranes via a Transblot Turbo (Biorad) using the mixed MW (SNAP-25) or low MW (VAMP-2) programs. Membranes were blocked for 1 h at room temperature with 5% low fat milk/PBS-Tween and then incubated with either anti-SNAP-25 (Sigma-Aldrich S9684 1:4000) or anti-VAMP-2 (Eurogentec custom made 1:500) primary antibody overnight at 4 °C. Membranes were washed 3 times in PBS-Tween and incubated with anti-rabbit-HRP secondary antibody for 1 h at room temperature. Membranes were washed for 3 × 5 min in PBS-Tween, then developed with SuperSignal West Dura or West Femto chemiluminescent substrate and visualized using a Syngene PXi system. Band densitometry was analysed using Genetools software and % protein cleavage was determined using the ratio of the full-length protein to the cleaved product for both SNAP-25 and VAMP-2.

### 4.11. Mouse Phrenic Nerve Hemidiaphragm Assay

The mouse phrenic nerve hemidiaphragm (mPNHD) assay is an ex vivo model used to measure the effect of botulinum neurotoxin (BoNT) at its in vivo target, the neuromuscular junction. Phrenic nerve hemidiaphragm tissue, from male CD1 mice (Charles River Laboratories, Margate, Kent, UK), was incubated in a 10 mL tissue bath (emkaBATH4 Tissue Bath System, emka Technologies, Paris, France) containing Krebs-Henseleit buffer (118 mM NaCl, 1.2 mM MgSO_4_, 11 mM Glucose, 4.7 mM KCl, 1.2 mM KH_2_PO_4_, 2.5 mM CaCl_2_, 25 mM NaHCO_3_, pH 7.5) gassed with 95% O_2_/5% CO_2_. The diaphragm muscle was indirectly stimulated (via the phrenic nerve) using 10 V, 1 Hz, 0.2 ms stimulation and resultant muscle contractions were recorded using an isometric force transducer (emka Technologies). Following a period of stabilization, 1 mL of 10× BoNT/A1, BoNT/F1 or BoNT/FA (final concentration 100 pM) was added to the tissue bath and electrical stimulation continued until the diaphragm muscle contraction was ablated. The decrease in contraction following toxin addition was calculated as a percentage of the contraction just before toxin addition and a four-parameter logistic curve fitted to the data using GraphPad Prism (GraphPad Prism version 6.07 for Windows, GraphPad Software, La Jolla, CA, USA, www.graphpad.com). From the curve fitted to the data, the time to 50% diaphragm paralysis (t_50_) was estimated. Statistical comparison between the mean t_50_ values for BoNT/A1, BoNT/F1 and BoNT/FA was performed using one-way ANOVA followed by Tukey’s multiple comparisons test (GraphPad Prism). Statistical significance was at *p* < 0.05.

### 4.12. Data Analysis

All results are expressed as mean ± standard error of mean of *n* independent experiments.

All dose-response data and hemidiaphragm paralysis traces were fitted to a four-parameter logistic equation using Graphpad Prism, version 6 (La Jolla, CA, USA).

Statistical differences between the three toxins were determined by one-way ANOVA, followed by Tukey’s multiple comparison post-hoc test where appropriate using GraphPad Prism. Significance was determined at the level *p* < 0.05.

## Supplementary Materials

The following are available online at http://www.mdpi.com/2072-6651/10/5/195/s1. Figure S1: Multiple sequence alignment of rBoNT/FA(0)-his, mrBoNT/FA(0)-his, and mrBoNT/FA-his, Figure S2: Primary amino acid sequence of mr-BoNT/FA-his. Light chain, LC/FA, amino acids shown in black, Figure S3: SNARE cleavage in rat cortical neurons panel (A) shows Western blot analysis of full-length and cleaved VAMP-2 from rat cortical neurons treated with nBoNT/F1 or mrBoNT/FA-his for 24 h.
