# Supplementary material for: Purification and Characterization of Recombinant Botulinum Neurotoxin Serotype FA, Also Known as Serotype H
Source: Toxins (Basel). 2018 May 11;10(5):195. doi: 10.3390/toxins10050195 (PMC5983251; doi:10.3390/toxins10050195)
Supplement: Supplementary file 1 [file toxins-10-00195-s001.pdf]

Supplementary Materials: Purification and Characterization of Recombinant Botulinum Neurotoxin Serotype FA, Also Known as Serotype H

Gavin Hackett, Kevin Moore, David Burgin, Fraser Hornby, Bryony Gray, Mark Elliott, Imran Mir and Matthew Beard

Supplemental Figure 1

Page S1

```
rBoNT-FA(0)-his   MPVVINSFNYDDPVNDNTIIYIRPPYYETSNTYFKAFQIMDNVWIIPERYRLGIDPSLFNPPVSLKAGSD   70
mrBoNT-FA(0)-his  MPVVINSFNYDDPVNDNTIIYIRPPYYETSNTYFKAFQIMDNVWIIPERYRLGIDPSLFNPPVSLKAGSD   70
mrBoNT-FA-his     MPVVINSFNYDDPVNDNTIIYIRPPYYETSNTYFKAFQIMDNVWIIPERYRLGIDPSLFNPPVSLKAGSD   70

rBoNT-FA(0)-his   GYFDPNYLSTNTEKNKYLQIMIKLFKRINSKPAGQILLEEIKNAIPYLGNSYTQEEQFTTNNRTVSFNVK  140
mrBoNT-FA(0)-his  GYFDPNYLSTNTEKNKYLQIMIKLFKRINSKPAGQILLEEIKNAIPYLGNSYTQEEQFTTNNRTVSFNVK  140
mrBoNT-FA-his     GYFDPNYLSTNTEKNKYLQIMIKLFKRINSKPAGQILLEEIKNAIPYLGNSYTQEEQFTTNNRTVSFNVK  140

rBoNT-FA(0)-his   LANGNIVQQMANLIIWGPGPDLTTNKTGGIIYSPYQSMEATPYKDGFGSIMTVEFSPEYATAFNDISIAS  210
mrBoNT-FA(0)-his  LANGNIVQQMANLIIWGPGPDLTTNKTGGIIYSPYQSMEATPYKDGFGSIMTVEFSPEYATAFNDISIAS  210
mrBoNT-FA-his     LANGNIVQQMANLIIWGPGPDLTTNKTGGIIYSPYQSMEATPYKDGFGSIMTVEFSPEYATAFNDISIAS  210

rBoNT-FA(0)-his   HSPSLFIKDPALILMHQLIYVLHGLYGTYITEYKITPNVVQSYMKVTKPITSAEFLTFGGRDRNIVPQSI  280
mrBoNT-FA(0)-his  HSPSLFIKDPALILMHQLIYVLHGLYGTYITEYKITPNVVQSYMKVTKPITSAEFLTFGGRDRNIVPQSI  280
mrBoNT-FA-his     HSPSLFIKDPALILMHELIHVLHGLYGTYITEYKITPNVVQSYMKVTKPITSAEFLTFGGRDRNIVPQSI  280

rBoNT-FA(0)-his   QSQLYNKVLSDYKRIASRLNKVNTATALINIDEFKNLYEWKYQFAKDSNGVYSVDLNKFEQLYKKIYSFT  350
mrBoNT-FA(0)-his  QSQLYNKVLSDYKRIASRLNKVNTATALINIDEFKNLYEWKYQFAKDSNGVYSVDLNKFEQLYKKIYSFT  350
mrBoNT-FA-his     QSQLYNKVLSDYKRIASRLNKVNTATALINIDEFKNLYEWKYQFAKDSNGVYSVDLNKFEQLYKKIYSFT  350

rBoNT-FA(0)-his   EFNLAYEFKIKTRLGYLAENFGPFYLPNLLDDSIYTEVDGFNIGALSINYQGQNIGSDINSIKKLQGQGV  420
mrBoNT-FA(0)-his  EFNLAYEFKIKTRLGYLAENFGPFYLPNLLDDSIYTEVDGFNIGALSINYQGQNIGSDINSIKKLQGQGV  420
mrBoNT-FA-his     EFNLAYEFKIKTRLGYLAENFGPFYLPNLLDDSIYTEVDGFNIGALSINYQGQNIGSDINSIKKLQGQGV  420

rBoNT-FA(0)-his   VSRVVRLCSNSN-TKN--S---LCITVNNRDLFFIASQESYGENTINTYKEIDDTTTLDPSFEDILDKVI  484
mrBoNT-FA(0)-his  VSRVVRLCKSVIPRKGTKAPPRLCITVNNRDLFFIASQESYGENTINTYKEIDDTTTLDPSFEDILDKVI  490
mrBoNT-FA-his     VSRVVRLCKSVIPRKGTKAPPRLCITVNNRDLFFIASQESYGENTINTYKEIDDTTTLDPSFEDILDKVI  490

rBoNT-FA(0)-his   LNFNEQVIPQMPNRNVSTDIQKDNYIPKYDYNRTDIIDSYEVGRNYNTFFYLNAQKFSPNESNITLTSSF  554
mrBoNT-FA(0)-his  LNFNEQVIPQMPNRNVSTDIQKDNYIPKYDYNRTDIIDSYEVGRNYNTFFYLNAQKFSPNESNITLTSSF  560
mrBoNT-FA-his     LNFNEQVIPQMPNRNVSTDIQKDNYIPKYDYNRTDIIDSYEVGRNYNTFFYLNAQKFSPNESNITLTSSF  560
```

Supplemental Figure 1

Page S2

```
rBoNT-FA(0)-his    DTGLLEGSKVYTFFSSDFINNINKPVQALLFIEWVKQVIRDFTTEATKTSTVDKLKDISLVVPYIGLALN   624
mrBoNT-FA(0)-his   DTGLLEGSKVYTFFSSDFINNINKPVQALLFIEWVKQVIRDFTTEATKTSTVDKLKDISLVVPYIGLALN   630
mrBoNT-FA-his      DTGLLEGSKVYTFFSSDFINNINKPVQALLFIEWVKQVIRDFTTEATKTSTVDKLKDISLVVPYIGLALN   630

rBoNT-FA(0)-his    IGDEIYKQHFAEAVELVGAGLLLEFSPEFLIPTLLIFTIKGYLTGSIRDKDKIIKTLDNALNVRDQKWKE   694
mrBoNT-FA(0)-his   IGDEIYKQHFAEAVELVGAGLLLEFSPEFLIPTLLIFTIKGYLTGSIRDKDKIIKTLDNALNVRDQKWKE   700
mrBoNT-FA-his      IGDEIYKQHFAEAVELVGAGLLLEFSPEFLIPTLLIFTIKGYLTGSIRDKDKIIKTLDNALNVRDQKWKE   700

rBoNT-FA(0)-his    LYRWVVSKWLTTINTQFNKRKEQMYKALKNQATAIKKIIENKYNNYTTDEKSKIDSSYNINEIERTLNEK   764
mrBoNT-FA(0)-his   LYRWVVSKWLTTINTQFNKRKEQMYKALKNQATAIKKIIENKYNNYTTDEKSKIDSSYNINEIERTLNEK   770
mrBoNT-FA-his      LYRWVVSKWLTTINTQFNKRKEQMYKALKNQATAIKKIIENKYNNYTTDEKSKIDSSYNINEIERTLNEK   770

rBoNT-FA(0)-his    INLAMKNIEQFITESSIAYLINIINNETIQKLKSYDDLVRRYLLGYIRNHSSILGNSVEELNSKVNNHLD   834
mrBoNT-FA(0)-his   INLAMKNIEQFITESSIAYLINIINNETIQKLKSYDDLVRRYLLGYIRNHSSILGNSVEELNSKVNNHLD   840
mrBoNT-FA-his      INLAMKNIEQFITESSIAYLINIINNETIQKLKSYDDLVRRYLLGYIRNHSSILGNSVEELNSKVNNHLD   840

rBoNT-FA(0)-his    NGIPFELSSYTNDSLLIRYFNKNYGELKYNCILNIKYEMDRDKLVDSSGYRSRINIGTGVKFSEIDKNQV   904
mrBoNT-FA(0)-his   NGIPFELSSYTNDSLLIRYFNKNYGELKYNCILNIKYEMDRDKLVDSSGYRSRINIGTGVKFSEIDKNQV   910
mrBoNT-FA-his      NGIPFELSSYTNDSLLIRYFNKNYGELKYNCILNIKYEMDRDKLVDSSGYRSRINIGTGVKFSEIDKNQV   910

rBoNT-FA(0)-his    QLSNLESSKIEVILNNGVIYNSMYENFSTSFWIRIPKYFRNINNEYKIISCMQNNSGWEVSLNFSNMNSK   974
mrBoNT-FA(0)-his   QLSNLESSKIEVILNNGVIYNSMYENFSTSFWIRIPKYFRNINNEYKIISCMQNNSGWEVSLNFSNMNSK   980
mrBoNT-FA-his      QLSNLESSKIEVILNNGVIYNSMYENFSTSFWIRIPKYFRNINNEYKIISCMQNNSGWEVSLNFSNMNSK   980

rBoNT-FA(0)-his    IIWTLQDTEGIKKTVVFQYTQNINISDYINRWIFVTITNNRLSNSKIYINGRLINEESISDLGNIHASNN  1044
mrBoNT-FA(0)-his   IIWTLQDTEGIKKTVVFQYTQNINISDYINRWIFVTITNNRLSNSKIYINGRLINEESISDLGNIHASNN  1050
mrBoNT-FA-his      IIWTLQDTEGIKKTVVFQYTQNINISDYINRWIFVTITNNRLSNSKIYINGRLINEESISDLGNIHASNN  1050

rBoNT-FA(0)-his    IMFKLDGCRDPHRYIWIKYFNLFDKELNKKEIKDLYDNQSNSGILKDFWGDYLQYDKPYYMLNLYDPNKY  1114
mrBoNT-FA(0)-his   IMFKLDGCRDPHRYIWIKYFNLFDKELNKKEIKDLYDNQSNSGILKDFWGDYLQYDKPYYMLNLYDPNKY  1120
mrBoNT-FA-his      IMFKLDGCRDPHRYIWIKYFNLFDKELNKKEIKDLYDNQSNSGILKDFWGDYLQYDKPYYMLNLYDPNKY  1120
```

Supplemental Figure 1

Page S3

```
rBoNT-FA(0)-his    LDVNNVGIRGYMYLKGPRGRIVTTNIYLNSTLYMGTKFIIKKYASGNKDNIVRNNDRVYINVVVKNKEYR  1184
mrBoNT-FA(0)-his   LDVNNVGIRGYMYLKGPRGRIVTTNIYLNSTLYMGTKFIIKKYASGNKDNIVRNNDRVYINVVVKNKEYR  1190
mrBoNT-FA-his      LDVNNVGIRGYMYLKGPRGRIVTTNIYLNSTLYMGTKFIIKKYASGNKDNIVRNNDRVYINVVVKNKEYR  1190

rBoNT-FA(0)-his    LATNASQAGVEKILSAVEIPDVGNLSQVVVMKSENDQGIRNKCKMNLQDNNGNDIGFIGFHQFNNIAKLV  1254
mrBoNT-FA(0)-his   LATNASQAGVEKILSAVEIPDVGNLSQVVVMKSENDQGIRNKCKMNLQDNNGNDIGFIGFHQFNNIAKLV  1260
mrBoNT-FA-his      LATNASQAGVEKILSAVEIPDVGNLSQVVVMKSENDQGIRNKCKMNLQDNNGNDIGFIGFHQFNNIAKLV  1260

rBoNT-FA(0)-his    ASNWYNRQIGKASRTFGCSWEFIPVDDGWGESSLHHHHHHHHHH                            1298
mrBoNT-FA(0)-his   ASNWYNRQIGKASRTFGCSWEFIPVDDGWGESSLHHHHHHHHHH                            1304
mrBoNT-FA-his      ASNWYNRQIGKASRTFGCSWEFIPVDDGWGESSLHHHHHHHHHH                            1304
```

Figure S1. Multiple sequence alignment of rBoNT/FA(0)-his, mrBoNT/FA(0)-his, and mrBoNT/FA-his. Positions of the E227Q and H230Y catalytic site inactivating mutations, the S429 to L437 activation loop substitution, and C-terminal ten-histidine affinity tag, are boxed in grey.

MPVVINSFNYDDPVNDNTIIYIRPPYYETSNTYFKAFQIMDNVWIIPERYRLGIDPSLFNPPVSLKA
GSDGYFDPNYLSTNTEKNKYLQIMIKLFKRINSKPAGQILLEEIKNAIPYLGNSYTQEEQFTTNNRT
VSFNVKLANGNIVQQMANLIIWGPGPDLTTNKTGGIIYSPYQSMEATPYKDGFGSIMTVEFSPEYAT
AFNDISIASHSPSLFIKDPALILMHELIHVLHGLYGTYITEYKITPNVVQSYMKVTKPITSAEFLTF
GGRDRNIVPQSIQSQLYNKVLSDYKRIASRLNKVNTATALINIDEFKNLYEWKYQFAKDSNGVYSVD
LNKFEQLYKKIYSFTEFNLAYEFKIKTRLGYLAENFGPFYLPNLLDDSIYTEVDGFNIGALSINYQG
QNIGSDINSIKKLQGQGVVSRVVRLC$_{428}$KSVIPRKGTKAPPRL*C$_{444}$ITVNNRDLFFIASQESYGENT*
INTYKEIDDTTTLDPSFEDILDKVILNFNEQVIPQMPNRNVSTDIQKDNYIPKYDYNRTDIIDSYEV
GRNYNTFFYLNAQKFSPNESNITLTSSFDTGLLEGSKVYTFFSSDFINNINKPVQALLFIEWVKQVI
RDFTTEATKTSTVDKLKDISLVVPYIGLALNIGDEIYKQHFAEAVELVGAGLLLEFSPEFLIPTLLI
FTIKGYLTGSIRDKDKIIKTLDNALNVRDQKWKELYRWVVSKWLTTINTQFNKRKEQMYKALKNQAT
AIKKIIENKYNNYTTDEKSKIDSSYNINEIERTLNEKINLAMKNIEQFITESSIAYLINIINNETIQ
KLKSYDDLVRRYLLGYIRNHSSILGNSVEELNSKVNNHLDNGIPFELSSYTNDSLLIRYFNKNYGEL
KYNCILNIKYEMDRDKLVDSSGYRSRINIGTGVKFSEIDKNQVQLSNLESSKIEVILNNGVIYNSMY
ENFSTSFWIRIPKYFRNINNEYKIISCMQNNSGWEVSLNFSNMNSKIIWTLQDTEGIKKTVVFQYTQ
NINISDYINRWIFVTITNNRLSNSKIYINGRLINEESISDLGNIHASNNIMFKLDGCRDPHRYIWIK
*YFNLFDKELNKKEIKDLY**DNQSNSGILKDFWGDYLQYDKPYYMLNLYDPNKYLDVNNVGIRGYMYLK***
GPRGRIVTTNIYLNSTLYMGTKFIIKKYASGNKDNIVRNNDRVYINVVVKNKEYRLATNASQAGVEK
ILSAVEIPDVGNLSQVVVMKSENDQGIRNKCKMNLQDNNGNDIGFIGFHQFNNIAKLVASNWYNRQI
GKASRTFGCSWEFIPVDDGWGESSLHHHHHHHHHH

Light chain, LC/FA
BoNT/F1 activation loop
Translocation domain, H_N/FA
Unknown function, H_{CN}/FA
Receptor binding domain, H_{CC}/FA
Affinity tag (His_{10})

Figure S2. Primary amino acid sequence of mrBoNT/FA-his. Light chain, LC/FA, amino acids shown in black. Substituted BoNT/F1 activation loop amino acids shown underlined. *Translocation domain, H_N/FA*, amino acids shown in grey italic. *Unknown function, H_{CN}/FA*, amino acids shown in black italic. **Receptor binding domain, H_{CC}/FA**, amino acids shown in bold black italic. Affinity tag (His_{10}), amino acids shown in grey.

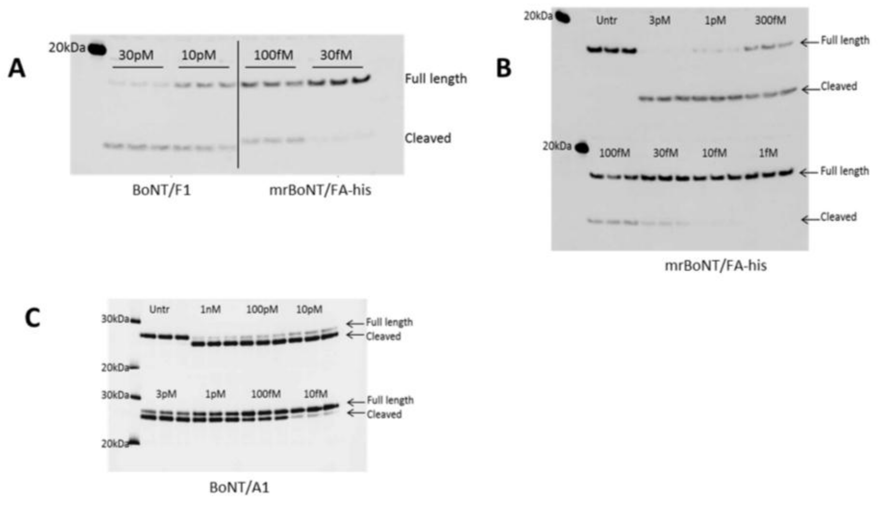

Figure S3. SNARE cleavage in rat cortical neurons panel (A) shows Western blot analysis of full-length and cleaved VAMP-2 from rat cortical neurons treated with nBoNT/F1 or mrBoNT/FA-his for 24 hours. Panel (B) shows Western blot analysis of full-length and cleaved VAMP-2 from rat cortical neurons treated with a concentration range of mrBoNT/FA-his for 24 hours. Panel (C) shows Western blot analysis of full-length and cleaved SNAP-25 from rat cortical neurons treated with a concentration range of nBoNT/A1 for 24 hours.
